# Disability-Adjusted Life-Years Associated With Intracerebral Hemorrhage and Secondary Injury

**DOI:** 10.1001/jamanetworkopen.2021.15859

**Published:** 2021-07-19

**Authors:** David Haupenthal, Joji B. Kuramatsu, Bastian Volbers, Jochen A. Sembill, Anne Mrochen, Stefanie Balk, Philip Hoelter, Hannes Lücking, Tobias Engelhorn, Arnd Dörfler, Stefan Schwab, Hagen B. Huttner, Maximilian I. Sprügel

**Affiliations:** 1 Department of Neurology, Friedrich-Alexander-Universität Erlangen-Nürnberg, Erlangen, Germany; 2 Department of Neuroradiology, Friedrich-Alexander-Universität Erlangen-Nürnberg, Erlangen, Germany

## Abstract

**Importance:**

Intracerebral hemorrhage (ICH) contributes significantly to the global burden of disease.

**Objective:**

To examine the association of ICH and secondary injury with disability-adjusted life-years (DALYs) for the individual patient.

**Design, Setting, and Participants:**

This cohort study was conducted using data from the Universitätsklinikum Erlangen Cohort of Patients With Spontaneous Intracerebral Hemorrhage study. Consecutive patients admitted to a single tertiary care center from January 1, 2006, to December 31, 2015, were included. The sample comprised patients with oral anticoagulation–associated ICH (OAC-ICH) or primary spontaneous ICH (non-OAC-ICH). Statistical analysis was conducted from October 1 to December 31, 2020.

**Exposures:**

ICH occurrence and secondary injury.

**Main Outcomes and Measures:**

DALYs, years of life lost (YLL), and years lived with disability (YLD) were analyzed by hematoma location, ICH volume, and secondary injury (ie, hematoma expansion [HE], intraventricular hemorrhage [IVH], and perihemorrhagic edema [PHE]).

**Results:**

Among 1322 patients with ICH, 615 (46.5%) were women and the mean (SD) age at hospital admission was 71 (13) years; ICH was associated with a mean (SD) of 9.46 (8.08) DALYs, 5.72 (8.29) YLL, and 3.74 (5.95) YLD. There were statistically significant differences in mean (SD) DALYs by extent of hematoma volume (< 10 mL ICH: 7.05 [6.79] DALYs; 10-30 mL ICH: 9.91 [8.35] DALYs; >30 mL ICH: 12.42 [8.47] DALYs; *P* < .001) and ICH location (deep location: 10.60 [8.35] DALYs; lobar location: 8.18 [7.63] DALYs; cerebellum: 8.14 [6.80] DALYs; brainstem: 12.63 [9.21] DALYs; *P* < .001). Regarding population-level disease burden of secondary injuries after ICH, there was a statistically significant difference in mean (SD) by injury type, with 0.94 (3.19) DALYs for HE, 2.45 (4.16) DALYs for IVH, and 1.96 (2.66) DALYs for PHE (*P* < .001) among the entire ICH cohort. Regarding individual-level exposure to secondary injuries after ICH, there were a mean (SD) 7.14 (6.62) DALYs for HE, 4.58 (4.75) DALYs for IVH, and 3.35 (3.28) DALYs for PHE among patients with ICH affected by secondary injuries.

**Conclusions and Relevance:**

These findings suggest that there is a high burden of disability associated with ICH and secondary injuries, and the findings may guide public health strategies. The study findings further suggest that IVH and PHE may be relevant for the overall outcome of patients with ICH, that DALYs may represent a viable outcome parameter for studies to evaluate treatment outcomes in ICH research, and that IVH and PHE may represent potential treatment targets.

## Key Points

**Question:**

What is the burden of intracerebral hemorrhage and secondary injury?

**Findings:**

In this cohort study of 1322 patients with intracerebral hemorrhage, the condition was associated with 9.46 disability-adjusted life-years, while perihemorrhagic edema and intraventricular hemorrhage were associated with increased disability compared with hematoma expansion in the overall cohort.

**Meaning:**

These findings suggest that intracerebral hemorrhage is associated with a high burden of disability, and these findings may guide public health strategies.

## Teaser

This cohort study examines the association of intracerebral hemorrhage and secondary injury with disability-adjusted life years.

## Introduction

The global burden of diseases is quantified at regular intervals using disability-adjusted life-years (DALYs).^1^ These are defined as the combination of years of life lost (YLL) owing to premature mortality and years lived with disability (YLD).^1^ Intracerebral hemorrhage (ICH) contributes significantly to the global burden of disease.^2,3^ However, the association of a single ICH event with DALYs for the individual patient has not been specified so far.

Several randomized controlled trials^4,5,6,7^ evaluated treatment options targeting secondary injury after ICH, such as hematoma expansion (HE), intraventricular hemorrhage (IVH), and perihemorrhagic edema (PHE). However, to our knowledge, these secondary injuries have never been compared against each other regarding their association with clinical outcomes. Therefore, it is still uncertain which parameter should be the primary treatment target in ICH research.

Functional outcome, quantified by the modified Rankin Scale (mRS), is usually defined as the primary end point in ICH studies.^4,5,6,7^ So far, to our knowledge, no single randomized trial has reported a significant treatment association measured by the mRS among patients with ICH.^8^ Given the disease severity and its enormous health care and economic implications, additional outcome parameters seem justified to identify and improve treatment options and decrease the burden of ICH disease. As a measure of disease burden, DALYs represent an obvious choice as alternative outcome parameter in ICH research. The purpose of the present study was to assess the association of ICH occurrence, hematoma location, and ICH volume with DALYs and to compare the association of secondary injury with DALYs by hematoma expansion, IVH, and PHE, thereby identifying the most relevant future treatment targets.

## Methods

Written informed consent for this cohort study was obtained from patients or legal representatives, and the study was approved by the local institutional review board at Friedrich-Alexander-University Erlangen-Nuremberg in Germany (115_17B). The study is reported following the Strengthening the Reporting of Observational Studies in Epidemiology (STROBE) reporting guideline.

Detailed methods of the Universitätsklinikum Erlangen Cohort of Patients With Spontaneous Intracerebral Hemorrhage (UKER-ICH; NCT03183167) cohort study have been published previously.^9,10^ In brief, consecutive patients with ICH admitted to the University Hospital Erlangen from January 1, 2006, to December 31, 2015, were included in a prospective, single-center institutional registry. Patients with ICH owing to secondary etiology, such as aneurysm, intratumoral hemorrhage, trauma, or arteriovenous malformation, were excluded. Analyses were conducted among patients with oral anticoagulation–associated ICH (OAC-ICH) and primary spontaneous ICH (non–OAC-ICH). Data on demographic characteristics, premorbid conditions, status at hospital admission, and laboratory and intrahospital parameters were assessed as previously published.^9,11^ High burden of cardiovascular disease was defined as history of ischemic stroke or transient ischemic attack (TIA) and congestive heart failure. Low burden of cardiovascular disease was defined as history of ischemic stroke or TIA, history of congestive heart failure, or none of these.

### Imaging

Hematoma characteristics and PHE were assessed on each imaging slice of all available imaging scans during patient hospital stays.^9,12^ We calculated PHE using a semiautomatic volumetric algorithm and defined PHE as the maximum edema volume among available imaging scans.^13,14^ Relative PHE was defined as the ratio of peak PHE volume to ICH surface area (calculated via the formula: ICH surface area = π^1/3^ × [6 × ICH volume]^2/3^).^12,15^ We calculated ICH volume using the ABC/2 method (A [greater hemorrhage diameter in the axial plane] times B [hemorrhage diameter at 90º to A in the axial plane] times C [number of computed tomography slices with hemorrhage], divided by 2) in case of round to ellipsoid ICH or ABC/3 method in case of irregularly shaped ICH.^16,17^ Hematoma enlargement was defined as a relative increase of more than 33% in ICH volume from initial imaging to follow-up imaging. Patients were divided into 3 groups by hematoma volume (ie, small ICH: <10 mL; medium ICH: 10-30 mL; and large ICH: >30 mL). The extent of IVH was assessed using the Graeb score; primary IVH was scored as deep ICH.^18^

### Outcome Measures

We calculated DALYs for each patient with ICH as the sum of YLL owing to premature mortality and YLD as the consequence of ICH impairment according to the current World Health Organization classification (Figure).^1^ We defined YLL as the difference between the patient’s age-specific life expectancy and age at death and YLD as the number of years lived with disability multiplied by a disability-weighting factor. Specific weighting factors for each degree of the mRS have been previously published and were used in this study (eTable 1 in the Supplement).^19^ For the UKER-ICH study, YLL and YLD were calculated using follow-up information on functional outcome and survival time. To perform accurate DALY assessment, YLD were specifically calculated for duration of hospital stay, from hospital discharge to 3 months after ICH diagnosis, from 3 months to 12 months after ICH diagnosis, and from 12 months after ICH diagnosis to death. The most recent functional status was applied for each time interval. In line with established methodology,^20^ the calculation did not include premorbid status, age-weighting, or future discount.

**Figure.**
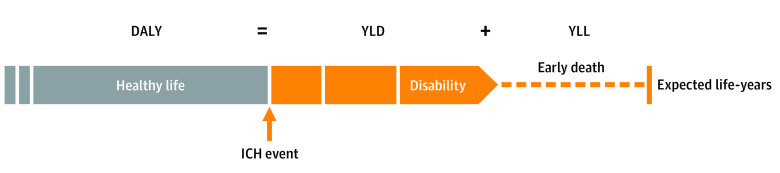
Disability-Adjusted Life-Years (DALYs) After Intracerebral Hemorrhage (ICH) Concept and calculation of years of life lost (YLL), years lived with disability (YLD), and DALYs are illustrated for patients with ICH. DALYs are defined as the sum of YLD and YLL owing to premature mortality as a result of the ICH event.

Additionally, YLL and YLD were assessed for the UKER-ICH study based on follow-up at 3 months and life expectancies derived from Federal Statistical Office of Germany data using calculation models (eMethods and eTable 2 in the Supplement).^19,21,22,23^ To investigate whether these previously published calculation models provided accurate DALY assessment, they were compared with the calculated values based on all available follow-up information (eMethods and eFigure 1 in the Supplement).

Data on functional outcome and mortality in the UKER-ICH study were assessed during hospital stay. These were evaluated by standardized mailed questionnaires or semistructured telephone interviews at 3 months and 12 months after the ICH event or retrieved from institutional databases in case of hospital readmission.^11,24^

### Statistical Analysis

Statistical analyses were performed using SPSS statistical software version 24.0 (IBM) from October 1 to December 31, 2020. We used 2-sided statistical tests and set the significance level to *P* = .05. Frequency distribution was determined using the Kolmogorov-Smirnov test. Categorical variables were compared using the χ^2^ test or Fisher exact test for proportions and given as total number and frequency in brackets. Continuous variables were compared using analysis of variance or Kurskal-Wallace test, respectively, and given as mean (SD).

For the calculation of YLL, YLD, and DALYs associated with secondary injury (attributable YLL [aYLL], attributable YLD [aYLD], and attributable DALYs [aDALYs] for extent of HE, IVH, and PHE), we multiplied attributable fractions by the overall YLL and YLD, respectively, for each secondary injury parameter. Attributable fractions were adjusted for relevant parameters associated with clinical outcomes after ICH (ie, age, National Institutes of Health Stroke Scale score, hematoma location, ICH volume, and secondary injury parameters [ie, HE volume, IVH extent, and PHE volume]).^25^ Mortality at 3 months after ICH was used for aYLL estimation, and good functional outcome (ie, mRS 0-3 at 3 months after ICH) was used for aYLD estimation. Calculation of aYLL, aYLD, and aDALYs was performed for the overall cohort (ie, population-level disease burden of secondary injuries) and for the subgroup of patients affected by secondary injury (ie, individual-level exposure to secondary injuries). Patients who were affected were defined by the presence of HE, IVH, and relevant PHE (ie, relative PHE, or ratio of PHE to ICH surface, of >1.5). We calculated aDALYs as the sum of aYLL and aYLD (eMethods in the Supplement).^26^

Studies in cerebrovascular diseases focus on clinical outcomes within 3 months after the index event and rarely obtain long-term outcomes.^27^ To evaluate, if DALYs may be determined in these studies, we compared DALY assessment (based on 3 months functional outcome only) with DALY calculation (based on information on long-term functional outcome and survival time, available for the UKER-ICH cohort).

## Results

Among 1322 patients with ICH, 615 (46.5%) were women and the mean (SD) age at hospital admission was 71 (13) years (Table 1; eFigure 2 in the Supplement). There were 587 patients with deep hematoma location (44.4%), 574 patients with lobar ICH (43.4%), 100 patients with cerebellar ICH (7.6%), and 61 patients with brainstem ICH (4.6%). Information on missing data is provided in eMethods and eAppendix in the Supplement.

**Table 1.** Clinical Characteristics of Patients by Hematoma Location

Characteristic	Patients, No. (%)				*P* value
	Deep location (n = 587)	Lobar location (n = 574)	Cerebellum (n = 100)	Brainstem (n = 61)	
Age, mean (SD), y	70 (12)	72 (13)	71 (12)	65 (14)	<.001
Women	247 (42.1)	281 (49.0)	56 (56.0)	31 (50.8)	.02
Men	340 (57.9)	293 (51.0)	44 (44.0)	30 (49.2)	.02
Prior comorbidities					
Hypertension	527 (89.8)	456 (79.4)	87 (87.0)	47 (77.0)	<.001
Prior ischemic stroke or TIA	133 (22.7)	96 (16.7)	24 (24.0)	9 (14.8)	.04
Prior hemorrhagic stroke or major bleeding	41 (7.0)	83 (14.5)	8 (8.0)	4 (6.6)	<.001
Congestive heart failure	91 (15.5)	70 (12.2)	12 (12.0)	6 (9.8)	.30
Admission status, median (IQR)					
NIHSS score	15 (8-28)	10 (4-21)	5 (3-24)	12 (5-29)	<.001
ICH score	2 (1-2)	1 (0-3)	2 (1-3)	2 (1-3)	<.001

Abbreviations: ICH, intracerebral hemorrhage; IQR, interquartile range; NIHSS, National Institutes of Health Stroke Scale; TIA, transient ischemic attack.

The Figure illustrates the concept and calculation of YLL, YLD, and DALYs for patients with ICH. Differences in outcome assessment between mRS and DALYs are outlined in examples of patients with ICH (Table 2). Conceptually, dichotomized end points of favorable outcome (eg, defined as mRS 0-3) did not account for the different clinical outcomes, while DALYs delineated these outcome differences.^28^

**Table 2.** DALYs and mRS in Patient Examples

Patient No.	Age, y	ICH		HE	IVH	mRS at 3 mo	Favorable outcome (mRS 0-3)	YLD	YLL	DALYs
		Location	Volume, mL							
1	70	Lobar location	5	No	No	1	Yes	1.08	0.61	1.69
2	70	Lobar location	5	Yes	No	2	Yes	2.78	2.43	5.21
3	65	Deep location	30	No	No	3	Yes	4.20	4.50	8.70
4	65	Deep location	30	No	Yes	4	No	6.52	7.35	13.87
5	65	Deep location	30	Yes	Yes	5	No	10.97	6.45	17.42

Abbreviations: DALY, disability-adjusted life-year; HE, hematoma enlargement; ICH, intracerebral hemorrhage; IVH, intraventricular hemorrhage; mRS, modified Rankin Scale; YLD, years lived with disability; YLL, years of life lost.

In the overall cohort, ICH was associated with a mean (SD) 5.72 (8.29) YLL, 3.74 (5.95) YLD, and 9.46 (8.08) DALYs. There were no statistically significant differences in DALYs between patients with non–OAC-ICH and those with OAC-ICH. There were more DALYs among patients with high burden of cardiovascular disease compared with patients with a low burden, although this increase was not statistically significant (eMethods and eAppendix in the Supplement).

### Burden of ICH by Hematoma Location

There were statistically significant differences by hematoma location in mean (SD) DALYs (deep location: 10.60 [8.35] DALYs; lobar location: 8.18 [7.63] DALYs; cerebellum: 8.14 [6.80] DALYs; brainstem: 12.63 [9.21] DALYs; *P* < .001) and mean (SD) YLD (deep location: 4.72 [7.06] YLD; lobar location: 2.69 [4.43] YLD; cerebellum: 3.31 [4.74] YLD; brainstem: 5.03 [6.87] YLD; *P* < .001) (Table 3). There were increased YLL among patients with brainstem ICH, but there were no statistically significant differences in YLL by hematoma location.

**Table 3.** Burden of Intracerebral Hemorrhage by Hematoma Location

Outcome	Mean (SD)				*P* value
	Deep location	Lobar location	Cerebellum	Brainstem	
Patients, No.	587	574	100	61	NA
Years of life lost	5.89 (8.39)	5.45 (8.06)	4.83 (7.30)	7.60 (10.63)	.18
Years lived with disability	4.72 (7.06)	2.69 (4.43)	3.31 (4.74)	5.03 (6.87)	<.001
Disability-adjusted life-years	10.60 (8.35)	8.18 (7.63)	8.14 (6.80)	12.63 (9.21)	<.001

Abbreviation: NA, not applicable.

### Burden of ICH by Extent of Hematoma Volume

There were statistically significant differences in mean (SD) DALYs by hematoma volume (small ICH: 7.05 [6.79] DALYs; medium ICH: 9.91 [8.35] DALYs; large ICH: 12.42 [8.47] DALYs; *P* < .001). There were also statistically significant differences by hematoma volume in mean (SD) YLL (small ICH: 3.21 [6.55] YLL; medium ICH: 5.52 [8.40] YLL; large ICH: 9.42 [9.04] YLL; *P* < .001) and mean (SD) YLD (small ICH: 3.84 [4.96] YLD; medium ICH: 4.39 [6.54] YLD; large ICH: 3.00 [6.56] YLD; *P* = .005).

### Burden of ICH Associated With Secondary Injury

The relevance of secondary injury pathways (ie, extent of HE, IVH and PHE) may be different for the individual patient with secondary injury vs the entire population, given that the frequency of these secondary injuries may vary substantially. We therefore analyzed the association between secondary injury and burden of ICH among patients who were affected (ie, had secondary injuries) and separately among the entire ICH cohort. The associations of secondary injury with DALYs, YLL, and YLD are provided in Table 4. Analyses were conducted among 720 patients with ICH and available PHE data. Patients with secondary injury were defined by presence of relevant HE (75 patients [10.4%]), IVH (386 patients [53.6%]), and relevant PHE (316 patients [43.9%]).

**Table 4.** Secondary Injury and Associated Burden of Intracerebral Hemorrhage

Patient group^a^	Mean (SD)^b^			*P* value
	HE	IVH	PHE	
aYLL				
Among patients who were affected	4.53 (6.37)	2.49 (3.96)	0.61 (1.05)	NA
Among overall patients	0.58 (2.62)	1.33 (3.15)	0.39 (0.76)	<.001
aYLD				
Among patients who were affected	2.61 (4.87)	2.09 (4.06)	2.74 (3.55)	NA
Among overall patients	0.36 (1.82)	1.12 (3.15)	1.58 (2.74)	<.001
aDALYs				
Among patients who were affected	7.14 (6.62)	4.58 (4.75)	3.35 (3.28)	NA
Among overall patients	0.94 (3.19)	2.45 (4.16)	1.96 (2.66)	<.001

Abbreviations: aDALY, attributable disability-adjusted life-year; aYLD, attributable years lived with disability; aYLL, attributable years of life lost; HE, hematoma enlargement; IVH, intraventricular hemorrhage; NA, not applicable; PHE, perihemorrhagic edema.

^a^ Among 720 patients with secondary injury, patients who were affected were defined by the presence of relevant HE (ie, relative increase of intracerebral hemorrhage volume >30%; 75 patients [10.4%]), IVH (386 patients [53.6%]), or relevant PHE (ie, relative perihemorrhagic edema, or ratio of PHE to intracerebral hemorrhage surface, of >1.5; 316 patients [43.9%]).

^b^ Analyses were adjusted for age, National Institutes of Health Stroke Scale score, deep intracerebral hemorrhage location, intracerebral hemorrhage volume, and secondary injury parameters (ie, HE volume, IVH extension [by Graeb score], and PHE volume).

For mean (SD) aDALYs, there were statistically significant differences among all patients (HE: 0.94 [3.19] aDALYs; IVH: 2.45 [4.16] aDALYs; PHE: 1.96 [2.66] aDALYs; *P* < .001) and differences among patients who were affected (HE: 7.14 [6.62] DALYs; IVH: 4.58 [4.75] aDALYs; PHE: 3.35 [3.28] aDALYs). For mean (SD) aYLL, there were statistically significant differences among all patients (HE: 0.58 [2.62] aYLL; IVH: 1.33 [3.15] aYLL; PHE: 0.39 [0.76] aYLL; *P* < .001) and differences among patients who were affected (HE: 4.53 [6.37] aYLL; IVH: 2.49 [3.96] aYLL; PHE: 0.61 [1.05] aYLL). For mean (SD) aYLD, there were statistically significant differences among all patients (HE: 0.36 [1.82] aYLD; IVH: 1.12 [3.15] aYLD; PHE: 1.58 [2.74] aYLD; *P* < .001) and differences among patients who were affected (HE: 2.61 [4.87] aYLD; IVH: 2.09 [4.06] aYLD; PHE: 2.74 [3.55] aYLD).

### DALY Assessment Using Short-Term Functional Outcome

We compared DALY assessment (based on 3 months functional outcome only) with DALY calculation (based on information on long-term functional outcome and survival time, available for the UKER-ICH cohort). The mean (SD) difference between DALY calculation and formula assessment was −0.66 (3.37) DALYs, and the 95% limits of agreement were between −7.27 DALYs and 5.95 DALYs. (eFigure 1 in the Supplement).

## Discussion

To our knowledge, this cohort study represents the first comprehensive assessment of DALYs associated with a single ICH event and secondary injury parameters. We found that ICH was associated with an increased burden of disability, notably in the subset of patients with brainstem and deep hematoma ICH location. We found that IVH and PHE, compared with HE, were outcome-relevant for most patients with ICH. The results of our study may guide public health strategies and improve the focus of ICH research toward the most relevant treatment targets. Furthermore, our findings suggest that DALYs may represent a viable outcome parameter that should be addressed by future ICH studies.

Defined as the combination of years of life lost owing to premature mortality and years lived with disability, DALYs appear to be the perfect measure of morbidity and mortality associated with intracerebral hemorrhage. However, the association of a single ICH event and DALYs for the individual patient have not been specified so far, to our knowledge, given that such individual patient data are not available for the Global Burden of Diseases Study.^1^ We found that ICH occurrence was associated with 9.5 DALYs, substantially more than the 5.9 DALYs associated with severe ischemic stroke.^21^ We found that DALYs increased with increased ICH volumes and were highest in the subset of patients with brainstem and deep hematoma ICH locations. Our findings suggest that public health strategies may reduce DALYs by 8.1 years for each cerebellar ICH, 8.2 years for each lobar ICH, 10.6 years for each deep ICH, and 12.6 years for each brainstem ICH prevented.

Regarding secondary injury pathways, HE, IVH, and PHE have been independently associated with functional outcome and mortality.^9,13,18^ However, to our knowledge, the extent of secondary injury by different parameters has never been sufficiently compared. Therefore, the most relevant parameter as primary treatment target remains unclear. We found that HE was associated with 7.1 DALYs among patients affected by HE. In contrast, among the overall cohort of patients with ICH, among whom relevant HE occurred in 10.4% of individuals, HE was associated with 0.9 DALYs, while IVH and PHE had more than 2-fold greater relevance for clinical outcomes in terms of DALYs. Additionally, PHE appeared most relevant for YLD among patients who were affected and patients with ICH overall. Our results may contribute to identifying the most relevant treatment targets in ICH research. Prevention of HE must be focused on patients with high risk of HE selected by radiological and clinical parameters.^9,29^ However, to improve outcomes for most patients with ICH, our findings suggest that future research efforts should be aimed at treatment of IVH and PHE.^6,30,31^

Regarding outcome assessment, DALYs measure functional impairment during the remaining lifetime after ICH, while mRS measures functional status at a single time. Therefore, DALYs represent a continuous rather than a dichotomized or ordinal outcome variable with certain statistical advantages vs mRS.^28^ We here found that DALYs delineated and accurately quantified clinical outcomes in ICH. We further found that DALYs could be calculated in ICH using mRS at 3 months after ICH event and life expectancies derived from Federal Statistical Office of Germany data. Therefore, our findings suggest that DALYs represent a viable outcome parameter in ICH research and could measure small treatment-associated outcomes even in underpowered studies, which may not be detected by mRS.^5,6,7,15,30,32,33,34,35,36,37^ Future studies should include DALYs as a secondary outcome parameter to identify and improve treatment options and finally decrease the burden of ICH disease.

### Limitations

Our study has several limitations. Relevant bias by patient selection may have influenced outcomes in our cohort given that patients with ICH due to secondary etiology, such as aneurysmal ICH, were excluded. Specific data on PHE were not available for the entire cohort, and we did not account for premorbid functional status. Although the applied method of attributable fraction assessment has been shown to provide estimation rather than precise calculation, the relevance of different secondary injury parameters should be sufficiently addressed and uncertainty remains.^26,38,39,40,41^ The benefits of DALYs as an outcome parameter could be largely attributable to the combination of premature mortality and disability. However, the global burden of disease is defined by this combined outcome parameter, which may help to identify small treatment-associated outcomes in future ICH research.

## Conclusions

This study provides data on DALYs among patients with ICH and secondary injury and may guide public health strategies. While we found that the occurrence of HE was associated with clinical outcomes among patients who were affected, IVH and PHE were associated with increased disability in the overall cohort compared with HE. These findings suggest that DALYs may represent a viable outcome parameter that may be applied in retrospective and prospective studies to evaluate treatment outcomes in ICH research.

## Notes

Supplement. eMethods. **eAppendix.** Missing Data and Exploratory Analysis **eTable 1.** Disability Weights for Modified Rankin Scale Levels **eTable 2.** Annual Mortality After Intracerebral Hemorrhage and Ischemic and Hemorrhagic Stroke by Functional Status on Modified Rankin Scale **eFigure 1.** Bland-Altman Analysis Comparing Disability-Adjusted Life-Years Measurement **eFigure 2.** Patient Flowchart eReferences
