# Supplementary material for: Disability-Adjusted Life-Years Associated With Intracerebral Hemorrhage and Secondary Injury
Source: JAMA Netw Open. 2021 Jul 19;4(7):e2115859. doi: 10.1001/jamanetworkopen.2021.15859 (PMC8290300; doi:10.1001/jamanetworkopen.2021.15859)
Supplement: Supplement. — eMethods. eAppendix. Missing Data and Exploratory Analysis eTable 1. Disability Weights for Modified Rankin Scale Levels eTable 2. Annual Mortality After Intracerebral Hemorrhage and Ischemic and Hemorrhagic Stroke by Functional Status on Modified Rankin Scale eFigure 1. Bland-Altman Analysis Comparing Disability-Adjusted Life-Years Measurement eFigure 2. Patient Flowchart eReferences [file jamanetwopen-e2115859-s001.pdf]

Supplemental Online Content

Haupenthal D, Kuramatsu JB, Volbers B, et al. Disability-adjusted life-years associated with intracerebral hemorrhage and secondary injury. *JAMA Netw Open*. 2021;4(7):e2115859. doi:10.1001/jamanetworkopen.2021.15859

eMethods.

eAppendix. Missing Data and Exploratory Analysis

eTable 1. Disability Weights for Modified Rankin Scale Levels

eTable 2. Annual Mortality After Intracerebral Hemorrhage and Ischemic and Hemorrhagic Stroke by Functional Status on Modified Rankin Scale

eFigure 1. Bland-Altman Analysis Comparing Disability-Adjusted Life-Years Measurement

eFigure 2. Patient Flowchart

eReferences

This supplemental material has been provided by the authors to give readers additional information about their work.

© 2021 Haupenthal D et al. *JAMA Network Open.*

eMethods

Years of life lost (YLL) were determined by the difference between life expectancy and age at death. Life expectancy was assessed for each patient using age- and gender-specific mortality tables of the Federal Statistical Office [1]. Time of death was recorded in the UKER-ICH study (complete follow-up until death was available for 635/1322 [48.0%] of patients and additional 2399 patient-years of follow-up for patients without information on the specific time of death). We calculated hazard ratios (HR) for annual mortality in post-ICH years related to functional status - for each level of the modified Rankin Scale (mRS) at 12 months after ICH - among ICH patients in the UKER-ICH study with complete follow-up until death. Calculated HRs for ICH patients of the UKER-ICH study were similar to stroke patients derived from the Athens Stroke Registry [2]. We referred to the Athens Stroke registry, as hazard ratios were reported for survivors at 3 months, ICH patients were included and there were no significant differences in the reported survival after 90 days among ischemic and hemorrhagic stroke patients [2]. For example, the UK Lothian Cohort Study and the Swedish Riks-Stroke Cohort Study reported hazard ratios for survivors at 6 months and did not include ICH patients [3]. HRs for annual mortality rates according to specific mRS scores were compared to a score of 0.

Years lived with disability (YLD) were determined by the duration of disability (number of years disabled) weighted by the level of disability. Disability weights ranging from 0 (normal) to 1 (dead) for functional status according to the modified Rankin Scale were applied. YLD were specifically calculated for the duration of hospital stay, the time between hospital discharge and 3 months after ICH, the time between 3 months and 12 months after ICH and the time between 12 months and death. The most recent functional status was applied for each time interval, functional improvement between 3 and 12 months after ICH was addressed by applying the functional status at 3 months between 3 and 6 months after ICH and the improved functional status at 12 months between 6 months and 12 months after ICH.

Attributable fraction (AF), defined as the proportion of disease attributed to exposure, was assessed using the equation AF=(OR-1)/OR, whereas odds ratios (OR) were adjusted for relevant parameters associated with clinical outcome after ICH (age, NIHSS, hematoma location, ICH volume and secondary injury parameters [hematoma enlargement volume, intraventricular hemorrhage extent and PHE volume]) 7. OR for mortality (3 months after ICH) was used for aYLL estimation and OR for good functional outcome (mRS 0-3 at 3 months after ICH) for aYLD estimation. Although this method of AF assessment has been shown to represent an estimation rather than a precise calculation of the attributable fraction, it allows to address the relevance of different secondary injury parameters 8-12.

To evaluate the influence of comorbidities on disease burden, we performed additional analyses regarding DALYs in the subgroups of non-OAC-ICH and OAC-ICH patients and according to history of CVD, i.e. history of ischemic stroke/TIA (IS) and/or history of congestive heart failure (CHF).

© 2021 Haupenthal D et al. *JAMA Network Open.*

eAppendix. Missing Data and Exploratory Analysis

Regarding anticoagulation at ICH onset, there were no significant differences in DALYs between non-OAC-ICH and OAC-ICH patients (non-OAC-ICH: 9.57 [±8.28] years versus OAC-ICH: 8.97 [±7.15] years; P=.29). Regarding history of cardiovascular diseases (CVDs), there were no significant differences in DALYs regarding history of ischemic stroke/TIA (non-IS: 9.42 [±8.39] years versus IS: 9.61 [±6.71] years; P=.73) or history of congestive heart failure (non-CHF: 9.44 [±8.12] years versus CHF: 9.58 [±7.87] years; P=.83). DALYs were higher in patients with high burden of CVD (defined as history of IS and CHF), although these differences were not statistically significant (low burden of CVD: 9.42 [±8.11] years versus high burden of CVD: 10.65 [±7.07] years; P=.33).

Missing data on demographic, in-hospital parameters and mortality rate at 3 months were 0/1322 (0.0%) for age, 0/1322 (0.0%) for sex, 3/1322 (0.2%) for hypertension, 9/1322 (0.7%) for prior ischemic stroke/TIA, 9/1322 (0.7%) for prior hemorrhagic stroke/major bleeding, 8/1322 (0.6%) for congestive heart failure, 0/1322 (0.0%) for NIHSS, 10/1322 (0.8%) for ICH score and 72/1322 (5.4%) for mortality at 3 months.

© 2021 Haupenthal D et al. *JAMA Network Open.*

eTable 1. Disability Weights for Modified Rankin Scale Levels[6]

mRS level	Disability weight
mRS 0	0.000
mRS 1	0.046
mRS 2	0.212
mRS 3	0.331
mRS 4	0.652
mRS 5	0.944

DALYs have previously been assessed in ischemic stroke patients for cohorts with follow-up information at 3 months only [6]. To evaluate the measurment of DALYs in intracerebral hemorrhage patients, we compared assessed DALYs (using the estimation formula and 3 months functional outcome only) to calculated DALYs (using follow-up information on functional outcome and survival time) in the UKER-ICH cohort.

© 2021 Haupenthal D et al. *JAMA Network Open.*

eTable 2. Annual Mortality After Intracerebral Hemorrhage and Ischemic and Hemorrhagic Stroke by Functional Status on Modified Rankin Scale

mRS level	HR from the UKER-ICH cohort	HR from the Athens Stroke Registry
mRS 0	1.0	1.0
mRS 1	1.10	1.18
mRS 2	1.35	1.55
mRS 3	2.21	1.80
mRS 4	3.18	2.57
mRS 5	5.61	5.72

As decided by the WHO in 2012 and applied in previous studies on ischemic stroke, we did not use discounting and age-weighting in the current study [4, 5].

YLD = T x DW

YLD = Years Lived with Disability; T=duration of disability; DW= Disability Weight

© 2021 Haupenthal D et al. *JAMA Network Open.*

eFigure 1. Bland-Altman Analysis Comparing Disability-Adjusted Life-Years Measurement

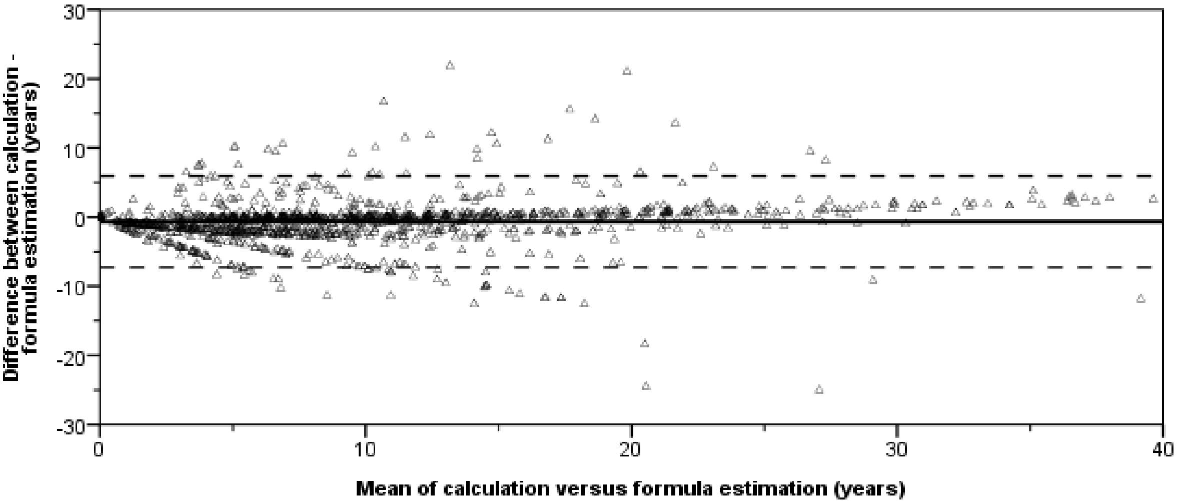

DALY calculation using information on functional outcome and survival time is compared *versus* DALY assessment using estimation formula and 3 months function outcome. Evaluation of bias revealed a mean difference of -0.66 (SD 3.37, solid line) DALYs (calculation – formula assessment). Assessment of DALYs provided accurate DALY measurement compared to DALY calculation using an accuracy threshold of ±6.61 DALYs (dotted lines).

© 2021 Haupenthal D et al. *JAMA Network Open.*

eFigure 2. Patient Flowchart

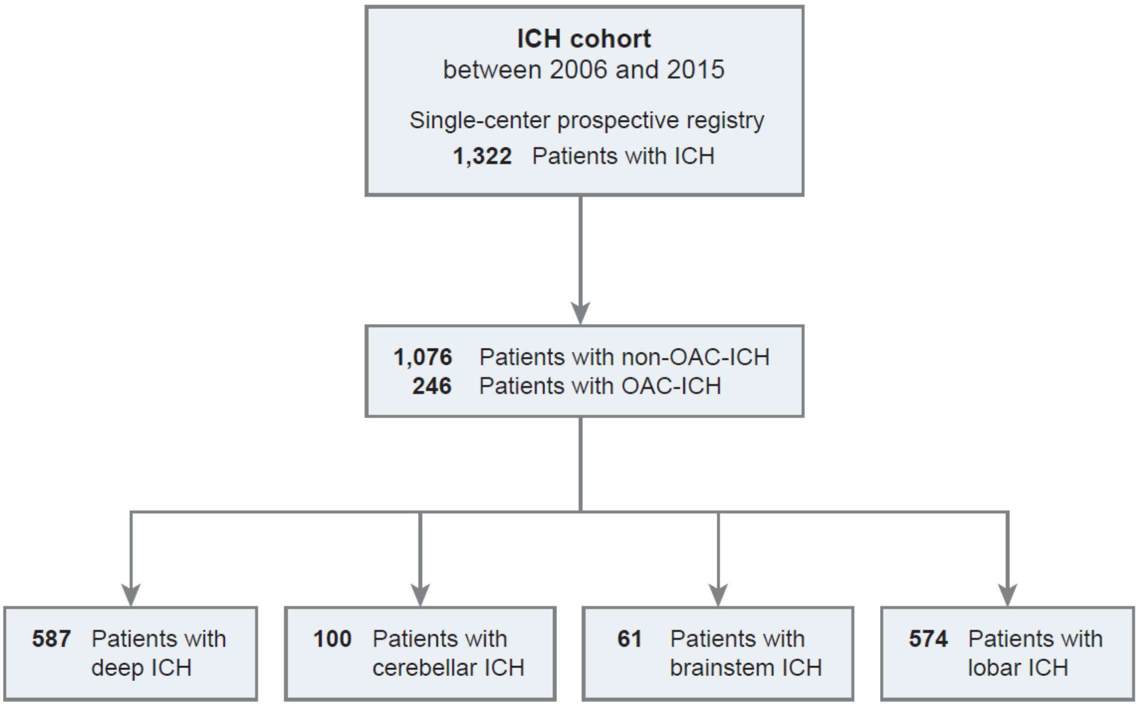

© 2021 Haupenthal D et al. *JAMA Network Open.*

eReferences

1. Bundesamt S. Kohortensterbetafeln für Deutschland. *published online June 23, 2017*. 2017;https://www.destatis.de/DE/Themen/Gesellschaft-Umwelt/Bevoelkerung/Sterbefaelle-Lebenserwartung/Publikationen/Downloads-Sterbefaelle/kohortensterbetafeln-5126101179004.pdf?__blob=publicationFile(Accessed November 05, 2020)
2. Huybrechts KF, Caro JJ, Xenakis JJ, Vemmos KN. The prognostic value of the modified Rankin Scale score for long-term survival after first-ever stroke. Results from the Athens Stroke Registry. *Cerebrovasc Dis*. 2008;26(4):381-7.
3. Slot KB, Berge E, Dorman P, Lewis S, Dennis M, Sandercock P. Impact of functional status at six months on long term survival in patients with ischaemic stroke: prospective cohort studies. *Bmj*. 2008;336(7640):376-9.
4. Meretoja A, Keshtkaran M, Saver JL, et al. Stroke thrombolysis: save a minute, save a day. *Stroke*. 2014;45(4):1053-8.
5. Murray CJ, Vos T, Lozano R, et al. Disability-adjusted life years (DALYs) for 291 diseases and injuries in 21 regions, 1990-2010: a systematic analysis for the Global Burden of Disease Study 2010. *Lancet*. 2012;380(9859):2197-223.
6. Hong KS, Saver JL. Quantifying the value of stroke disability outcomes: WHO global burden of disease project disability weights for each level of the modified Rankin Scale. *Stroke*. 2009;40(12):3828-33.
7. Mandava P, Murthy SB, Shah N, Samson Y, Kimmel M, Kent TA. Pooled analysis suggests benefit of catheter-based hematoma removal for intracerebral hemorrhage. *Neurology*. 2019;92(15):e1688-e1697.
8. Benichou J. A review of adjusted estimators of attributable risk. *Stat Methods Med Res*. 2001;10(3):195-216.
9. Cole P, MacMahon B. Attributable risk percent in case-control studies. *Br J Prev Soc Med*. 1971;25(4):242-4.
10. Gefeller O. Comparison of adjusted attributable risk estimators. *Stat Med*. 1992;11(16):2083-91.
11. Morgenstern H. Uses of ecologic analysis in epidemiologic research. *Am J Public Health*. 1982;72(12):1336-44.
12. Greenland S, Morgenstern H. *Morgenstern Corrects a Conceptual Error*. Am J Public Health. 1983 Jun;73(6):703-4.

© 2021 Haupenthal D et al. *JAMA Network Open.*
